# Influence of Thermal Contact Resistance of Aluminum Foams in Forced Convection: Experimental Analysis

**DOI:** 10.3390/ma10080907

**Published:** 2017-08-05

**Authors:** Stefano Guarino, Giovanni Di Ilio, Simone Venettacci

**Affiliations:** Department of Engineering, University of Rome ‘Niccolò Cusano’, Via Don Carlo Gnocchi 3, 00166 Roma, Italy; giovanni.diilio@unicusano.it (G.D.L.); simone.venettacci@unicusano.it (S.V.)

**Keywords:** open cell metal foams, heat transfer enhancement, contact resistance, forced convection

## Abstract

In this paper, the heat transfer performances of aluminum metal foams, placed on horizontal plane surface, was evaluated in forced convection conditions. Three different types of contacts between the sample and the heated base plate have been investigated: simple contact, brazed contact and grease paste contact. First, in order to perform the study, an ad hoc experimental set-up was built. Second, the value of thermal contact resistance was estimated. The results show that both the use of a conductive paste and the brazing contact, realized by means of a copper electro-deposition, allows a great reduction of the global thermal resistance, increasing de facto the global heat transfer coefficient of almost 80%, compared to the simple contact case. Finally, it was shown that, while the contribution of thermal resistance is negligible for the cases of brazed and grease paste contact, it is significantly high for the case of simple contact.

## 1. Introduction

Open-cell metal foams are a relatively recent class of materials that have been becoming popular in many research fields. Such materials are characterized by interesting properties from a structural as well as a thermal point of view. In particular, metal foams possess lightweight structure, high surface area-to-volume ratio and they are able to enhance flow mixing and increase turbulence. These features make them suitable for a wide range of heat transfer applications [1,2].

In light of growing industrial interest towards metal foams, several studies have been carried out in order to model metal foams structure and characterize their thermal behaviour. Comprehensive reviews of thermal models for metal foams can be found in [3,4]. Many studies focus on the design and optimization of manufacturing processes [5,6,7,8], while others are aimed to define and determine the metal foams thermal properties [4,9,10], In particular, Bhattacharya et al. [9] conducted analytical and experimental investigation in order to determine several properties of metal foams, such as: effective thermal conductivity, permeability and inertial coefficient. In their work, they introduce a theoretical model in which the complex interconnected array of fibres characterizing commercially available metal foams is represented as a two-dimensional array of hexagon cells. Another model is the one proposed by M. Bai et al. [11], which describes the metal foam geometries by adopting diamond-shape unit cells. By employing this model, the authors estimate the heat transfer capabilities of a foamed channel, obtaining good agreement with results from numerical simulations.

Furthermore, in the literature, there exists a significant body of research aimed at studying the heat transfer performance of metal foams in natural convection. Some of these works are conducted by modelling the involved phenomena via local thermal equilibrium [12,13], while few studies are based on the local thermal non equilibrium approach [14,15,16]. Among the others, we mention the experimental works of Bhattacharya et al. [17] and Barbieri et al. [18], who investigate the buoyancy-induced convection in copper and aluminum metal foams, demonstrating that the thermal transmission of heated surfaces can be enhanced by the use of such materials.

As far as the study of metal foams in forced convection is concerned, several works have been developed in literature. Kim et al. [19] conducted an experimental study on forced convection for aluminum metal foam placed in an asymmetrically heated channel. By testing samples of different permeability, they demonstrated that the friction factor is higher for foams of lower permeability. Calmidi et al. [10] carried out a numerical and experimental work on high porosity aluminum metal foams in forced convection. As far as the numerical analysis is concerned, they modelled the energy transport via local thermal non-equilibrium. As a result, they found that the transport phenomena are driven by the conduction through the solid phase.

Another relevant experimental study was made by Mancin et al. [20], which analyzed the behaviour of aluminum and copper metal foams in forced convection. Taking into consideration samples of different heights, pores per linear inch (PPI) and porosity, they defined several performance parameters and they developed two correlations for heat transfer coefficient and pressure drop. In conclusion, they found that, due to their conductivity values, copper foams exhibit more efficient thermal performance than the aluminum ones. Moreover, Boomsma et al. [21] studied the thermal performance of compact heat exchangers made by open-cell aluminum foam. Their analysis showed that such devices are characterized by a thermal resistance that is more than two times lower than the one related to the commercially available heat exchangers. Compact heat exchangers performances were also studied by Zhao et al. [22]. Through wind tunnel experiments, they estimated the heat transfer and the pressure drop, comparing and validating their results with [10,17]. Kim et al. [23] conducted an experimental investigation on a simplified model of heat exchanger constituted by porous aluminum fins. They analyzed many samples of different permeability and porosity and they compared such porous fins with conventional fins in term of thermal performance. From their results, at relatively low Reynolds numbers, the values of friction factor are lower for the porous media. Huisseune et al. [24] performed two dimensional numerical simulations adopting the thermal non-equilibrium energy model on metal foam heat exchangers. They found that louvered fins heat exchangers exhibit better performance than heat exchanger made by foam, underlining the need of an optimization for such solutions. Hsieh et al. [25] carried out an experimental study to characterize the heat transfer behaviour of several heat sinks made of aluminum metal foams with different porosity and PPI. Zaho et al. and Lu et al. [22,26] analyzed the forced convection problem in a tube filled with a porous medium subjected to constant wall heat flux. Guarino et al. [16] conducted an experimental thermo-fluid dynamic analysis of different types of aluminum alloy foams. They determined the effects of operational parameters, foam pore size, air flux and temperature on the heat transfer coefficient and on the pressure drop. Ribeiro et al. [27] investigated the thermal-hydraulic performance of microchannel condensers with open-cell metal foams in order to enhance the air-side heat transfer. Through their analysis, they evaluated the influence of the pore density on the air-side heat transfer.

Due to high porosity and irregularities of metal foam surface, the contact area at the interface with a solid surface can be very small. This leads to high values of the Thermal Contact Resistance (TCR) at the metal foam/solid surface interface. The analyzed literature indicates that the TCR is usually neglected and/or included in the effective thermal conductivity [28,29].

Authors use different bonding methods in their study. In fact, metal foams are thermally connected with the heatsink by the use of different joining methods such as brazing, thermal compound (grease), epoxy resin or even by simply applying a load between the contact surfaces. Sadeghi et al. [30] showed that TCR is negligible when compared to the medium resistance for ideally brazed metal foams. Moreover, they analyzed the influence of TCR on thermal conductivity when a load is applied on the foam sample. Their results show a large contribution of TCR to the total thermal resistance especially for low values of the compressive load.

The influence of TCR on effective thermal conductivity and heat transfer coefficient is still an open issue. In fact, from the literature analysis it emerges that there is still a lack of knowledge regarding the influence of the joining method on the global heat transfer coefficient for metal foams.

The aim of this work was to investigate the effects due to the use of different types of contact between the foam and the heatsink on the thermal performance of aluminum foams in forced convection. In particular, three cases were considered: (i) simple contact; (ii) metal foam connected to the hot plate by a layer of thermal grease; (iii) metal foam brazed on the hot plate. The global thermal resistance was evaluated for all the cases analyzed. Moreover, both the thermal contact resistance and the thermal convective resistance contributions were estimated, thus providing a new insight on the impact of the contact solution on the thermal heat transfer for aluminum foams.

## 2. Experimental (Materials and Methods)

The first step of the experimentation was aimed to evaluate the heat transfer performances of aluminum metal foams placed on horizontal plane surface, heated from the bottom, in forced convection conditions. In particular, an experimental setup has been implemented in order to investigate the effects due to the thermal contact resistance between metal foam and heated plate on the global heat transfer coefficient U.

Cubic samples of 50 mm side have been used for all the cases analyzed. All the considered samples are obtained by cutting the same sheet of metal foam, which is made by aluminum alloy AlSi7Mg0.3 with nominal bulk thermal conductivity in the range 160–180 Wm^−1^K^−1^ at 20 °C. Therefore, the foam samples are characterized by nominally identical material properties and geometrical parameters. These are reported in Table 1.

**Figure 1:**
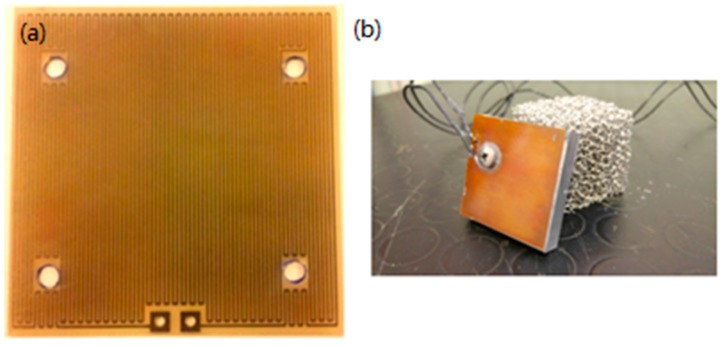
(**a**) Custom-made heater; (**b**) heater in contact with the aluminum plate.

In order to obtain a uniform distribution of the heat flow from the heater to the metal foam sample, a flat 10 mm thick aluminum plate, with same base dimension of the sample, has been placed between them. Therefore, to acquire the temperature at the interface between the sample and the plate, two K-type thermocouples have been placed into two horizontal holes, of 1.5 mm diameter, drilled into the sides of the plate, both to a distance of 1 mm from the foam base. However, in order to obtain an accurate representation of the thermal gradient across the foam base, the holes are located in two different positions: one in the middle of one side of the plate (20 mm deep) and the other in one of the corners (5 mm deep). At the bottom face of the plate a custom-made heater has been collocated. The heater has been realized by means of a printed circuit (whose thermal conductivity is about 3 W/mK), which function is to distribute the heat uniformly all over the bottom surface of the aluminum plate. In Figure 1 is clearly visible the labyrinth path of the circuit and the two edges where the wires are welded on.

### 2.1. Design of Experiments

Three different types of contacts between the foam and the heated plate have been analyzed:

(1) simple contact;
(2) heatsink compound paste;
(3) brazed contact.

For each case, different trials have been performed imposing the following array of differential temperature between the hot surface and the ambient: Δ*T* = 30, 50, 70, 90, 110 °C.

Despite brazed and grease contacts are not very affected by the pressure applied on the top of the foam, in case of simple contact, the contact resistance is instead strongly influenced by the pressure between metal foam sample and heated plate. In fact, in this case, an applied pressure deforms the pins in contact with the flat surface. Therefore, for the case of simple contact, it was necessary to ensure a certain load on the top of the sample. To accomplish this purpose, a 1 kg weight has been positioned onto the top surface providing a static, uniform and constant pressure, between the foam and the plate, for the entire duration of the experiment.

As far as case (2) is concerned, 3 g of a conductive grease (silicone thermal grease, $k_{paste}=5\;W/mK$, $\rho_{paste}=2.3\;g/cm^{3}$) are spread between the hot plate and the sample. The main role of thermal grease is to eliminate air gaps at interface in such a way to maximize the heat transfer between foam and plate. This aspect is crucial in metal foams, where the air gap between the hot plate and the metal foam is comparable to the characteristic length of the metal foam.

In case (3) the aluminum metal foam samples are brazed on the hot plate with a brazing alloy 60 Sn-40 Pb. However, since aluminum surfaces have a bad wettability by tin alloy, an electrodeposition of copper is made on both metal foam and hot plate surfaces, before brazing. In order to realize such a treatment, both the foam and the hot plate are preliminary sand blasted, in such a way to eliminate the oxides from the surfaces. Later, the aluminum foam and the aluminum plate are immerged in a solution of SO_4_ together with a copper electrode. The system is then connected to a DC generator [31].

**Figure 2:**
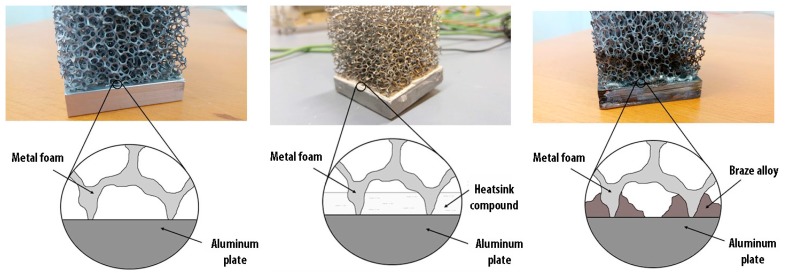
Schematic representation of the contact between foam and plate. From the left: (1) simple contact, (2) grease paste contact, (3) brazed contact.

In Figure 2 a schematic representation of the different types of contact studied is shown.

### 2.2. Setup Description

**Figure 3:**
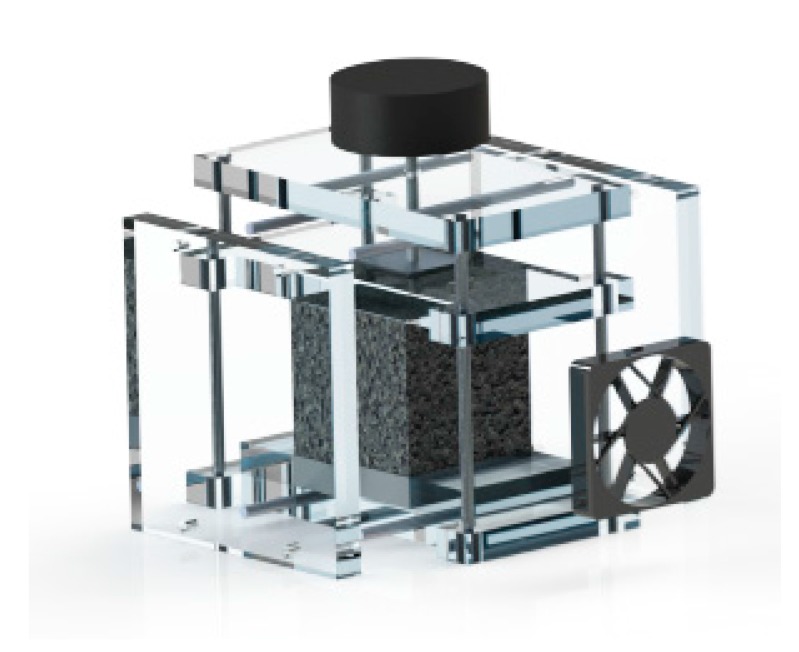
CAD modelling of the experimental set-up for the configuration with load.

The experimental setup was designed and optimized in such a way to allow the testing of each of the three different types of contact. To accomplish this purpose, a three-dimensional CAD model by using SolidWorks was preliminary realized. The final configuration is shown in Figure 3.

**Figure 4:**
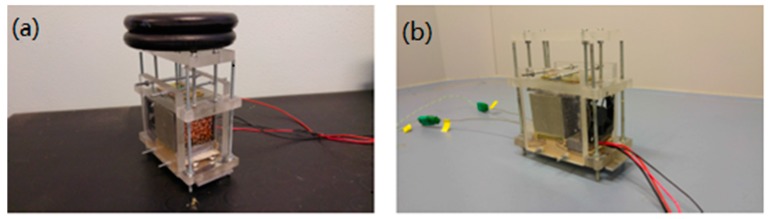
Experimental setup. (**a**): Housing with load; (**b**): housing without load.

The experimental setup consists in an open wind tunnel square cross-section, made of 10 mm thick Plexiglas (thermal conductivity is 0.25 W/mK), which air flow section measures 50 × 50 mm^2^ and it is extended for 100 mm. The size of the wind tunnel was chosen in such a way to ensure an unidirectional and sufficiently uniform air inflow. On the other hand, we assume the length of the outlet region not to be a critical parameter for the present analysis. Therefore, the overall size of the tunnel was finally designed on the base of constructive aspects. The lateral sides of the Plexiglas housing insulate the aluminum plate, while two other blocks of Plexiglas, having same thickness of the plate, are inserted at inlet and outlet of the tunnel, respectively, to ensure the insulation along the stream-wise direction. In addition, a thin layer of high temperature insulation paper is placed between the aluminum plate and the lateral sides of the Plexiglas housing. All the sides are bolted together in order to provide the same closing force for each trial. At the interior of each panel, a high temperature rubber insulator has been glued for avoiding air lack across the foam. The upper part of the rig is capable of sliding through guides and it has been used for distributing the load pressure for the case of simple contact. At the inner section of the case a 12 V DC vent is located for driving the air flow at a fixed speed of 1 m/s. The inlet temperature was monitored using two K-type thermocouples placed near the inlet. Figure 4 shows the test section, for the case of applied load and without load.

**Figure 5:**
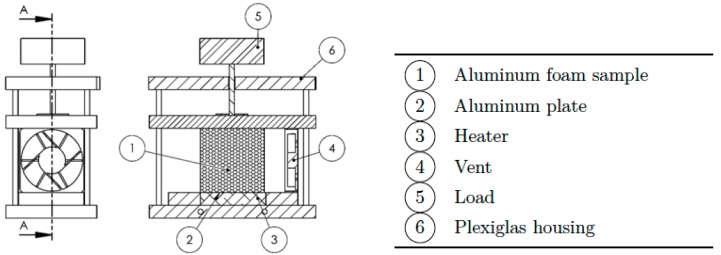
Schematic representation of the experimental set-up.

In Figure 5, where a schematic representation of the test case is shown, all the key elements of the setup are indicated.

### 2.3. Data Acquisition System

**Figure 6:**
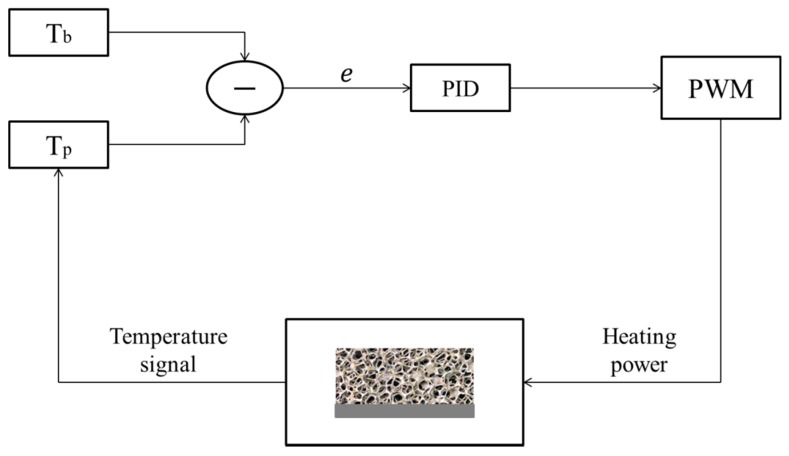
Schematic representation of the feedback system.

The target of our analysis was to evaluate the global heat transfer coefficient due to different kinds of contact. In order to accomplish this goal, a complex system of measurement has been developed to acquire, control and record the following parameters: (i) room temperature; (ii) aluminum plate temperature and (iii) power dissipated in the heater. In Figure 6 a schematic representation of the measurement system used for the experimentation is presented.

In order to guarantee stationary processes, a feedback system, based on a P.I.D. controller, was realized to keep the difference between ambient/inlet temperature and plate temperature around a constant value during the tests. In particular, the P.I.D. controller, implemented in LabView, drives a PWM generator which provides the required amount of thermal power to supply the heater in such a way to minimize the error function, defined as follows:

(1) $$e=|T_{b}-T_{p}|$$

where $T_{p}$ is the instantaneous temperature of the aluminum plate and $T_{b}$ is the foam base temperature, given by:

(2) $$T_{b}=T_{amb}+\Delta T$$

Moreover, the dissipated power Q due to Joule effect is calculated by the direct measurement of the current flowing through the heater circuit and the voltage drop. The current intensity was determined from the knowledge of a potential difference along a shunt resistance of 0.01 Ω. All the quantities are acquired by a National Instruments data acquisition.

As a general criterion, the data acquisition of the variables of interest, namely $T_{p}$, $T_{amb}$ and Q, is performed when the error function is e≅ 0.1 K.

### 2.4. Assembly Procedure

The assembly of the experimental setup represents the preliminary phase of each of the three cases analyzed. The assembly procedure has been designed in order to ensure repeatability and reproducibility of the tests.

**Figure 7:**
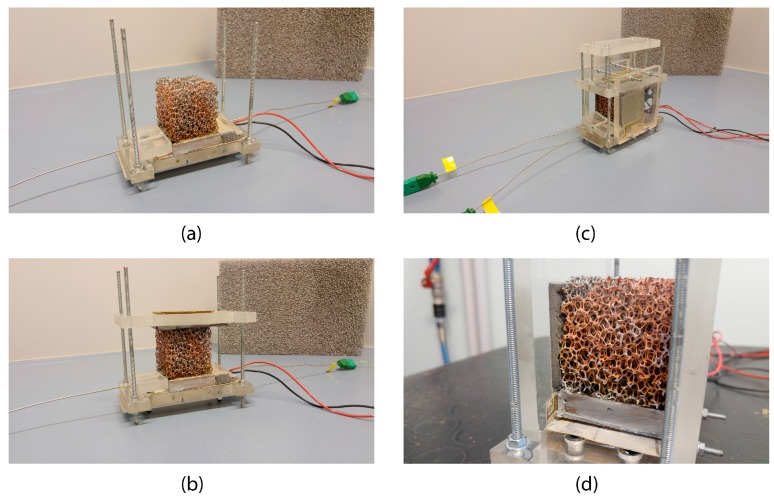
Phases of the assembly procedure; (**a**): First part of the assembly- Foam positioning; (**b**): Second part of the assembly-The upper part of the plexiglass house is positioned on the top of the foam; (**c**): Final phase of the assembly-The vent is positioned and the system is ready to be used; (**d**): A detail view of the foam insulating.

First of all, the foam sample is placed onto the aluminum plate which is positioned onto the housing base. Second, the thermocouples are inserted within the specific holes. The upper side of the Plexiglas housing is then shifted through four pins until contact with the top face of the foam occurs. For the case of simple contact, the weight is positioned on the top of this element. Later, the two lateral sides of the housing are bolted to the main structure. Finally, the vent is positioned at the inlet of the system. Figure 7 illustrates the different phases of the assembly procedure.

## 3. Uncertainty Analysis

The uncertainty of measurement is due to random as well as systematic errors that affect the following quantities: heat flow, temperature difference and physical dimensions. The heat losses through the insulation as well as conduction losses through the thermocouples and power supply wires were considered negligible. Thus, the uncertainty on heat flow value is due to uncertainty on the measurement of voltage drop along the heater, along the shunt resistance and on the value of the shunt resistance itself. The estimated inaccuracy $u_{Q}$ for the power input is 1.59%. Moreover, the inaccuracy in temperature measurement $u_{\Delta T}$ is 0.94% and it takes into account the resolution of data acquisition unit and thermocouple calibration. The base area of the sample is estimated with an uncertainty of 0.80%. Therefore, the overall relative uncertainty of the heat transfer coefficient can be computed as follows:

(3) $$\frac{u_{U}}{U}=\sqrt{{\left(\frac{u_{Q}}{Q}\right)}^{2}+{\left(\frac{u_{\Delta T}}{\Delta T}\right)}^{2}+{\left(\frac{u_{A_{b}}}{A_{b}}\right)}^{2}}$$

As a result, a value of $u_{U\%}=2.0\;\%$ was obtained.

## 4. Results

In order to characterize the thermal behaviour of the metal foams for different contact configurations, three parameters have been analyzed, that are: (1) the dissipated power, (2) the global heat transfer coefficient and (3) the thermal contact resistance. The experimental results have been compared to those available in literature.

The global heat transfer coefficient *U* takes into account both contributions of contact resistance and convective resistance of the system, and it is computed as follows:

(4) $$U=\frac{Q}{A_{b}\Delta T}$$

where $A_{b}$ is the base area of the sample, Q is the heat power input to the heater, and $\Delta T=\left(T_{b}-T_{amb}\right)$ is the difference between the average temperature of the base and of the ambient.

**Figure 8:**
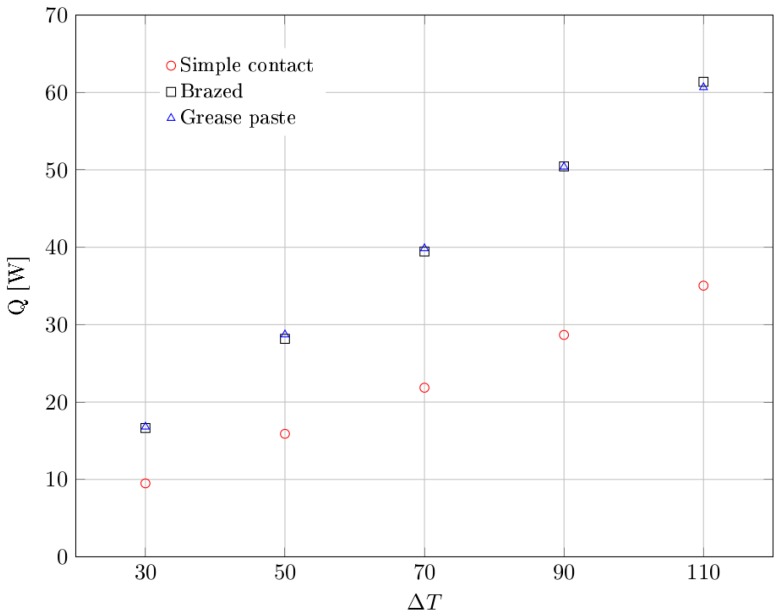
Dissipated heat power as a function of differential temperature, for each type of contact.

Next Figure 8 shows the average values for the amount of dissipated power as a function of differential temperature and type of contact. These values are also reported in Table 2.

From the experimental results it emerges that the dissipated power values for the cases of brazed contact and grease past contact are nearly the same in all the range of differential temperature taken into consideration. On the other hand, we notice a significant reduction of dissipated power when the simple contact between the sample and the hot plate is considered.

**Figure 9:**
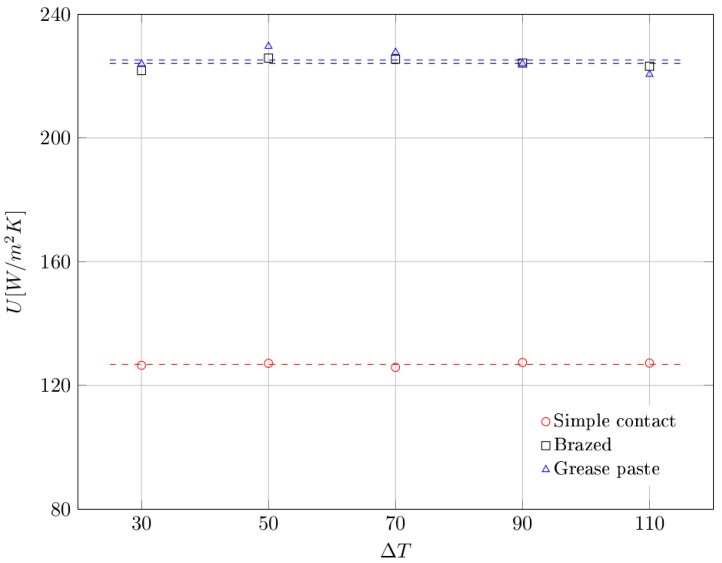
Global heat transfer coefficient as a function of differential temperature, for each type of contact. Dashed lines represent the average values.

In addition, we notice that the percentage difference of average dissipated power, between the case of simple contact and the others, remains fairly constant with the increasing of the differential temperature. Figure 9 illustrates the global heat transfer coefficient as a function of the differential temperature and type of contact. These values are also reported in Table 3.

As expected, it was found that the global heat transfer coefficient is not dependent from the imposed value of differential temperature but rather it depends only on the type of contact. In particular, it was observed that the average value of *U* is nearly the same for the cases of brazed (*U* = 224 W/m^2^K) and grease paste (*U* = 225 W/m^2^K) contact. For the simple contact case instead, there was a significant drop in the average value of global heat transfer coefficient (*U* = 127 W/m^2^K). These results demonstrate that the global heat transfer coefficient increases of about 80% when a brazing contact or a grease paste contact is used.

Experimental activities have therefore shown that is possible to arrive to the same results in the brazed contact and grease paste contact configurations. This is due to a considerable reduction of the contact resistance between the foam and the hot plate, compared to the simple contact. So the global heat transfer coefficient (*U*) becomes depending of the forced convection heat coefficient alone, thanks to a considerably greater conductive heat transfer section between the foam and the hot plate. The results also agree with the other literature results [32]. Fiedler el al. studied the effect of pressure contact on the thermal resistance. Fiedler found that only relatively small changes in the contact resistances can be observed over a certain value of the pressure, reaching a saturation value of the thermal exchange.

The evaluated value of U for the brazed contact case is in good agreement with the one obtained by application of the empirical correlation provided by Mancin et al. [33]. In this case, a value of 252.15 (W/m^2^K) was found. The percentage difference between this value and the one estimated in the present work is about 10%.

To further validate the results, a comparison has been made with the model proposed by Ghosh [34], in which an heat transfer correlation for high-porosity open-cell foams was developed. In particular, he proposed a correlation for the computation of the Nusselt number based on the fibre diameter of the foam. Such a model assumes porous media as a continuously connected structured with the total amount of heat being transferred from the foam base. For this reason, this model is suitable to approximate the case of brazed contact. By applying Ghosh’s formula, it was obtained a value of *Nu* ≈ 4.06. On the other hand, evaluating the Nusselt number by the following relation:

(5) $$Nu=\frac{d_{f}U}{k_{f}}$$

where $k_{f}$ = 0.026 W/mK is the thermal conductivity of the air, it was obtained, for the case of brazed contact, a value of Nu equal to 4.31. The two results are close to each other, confirming the validity of the global heat transfer coefficient predicted by the experimental analysis.

Results have been also analyzed in term of global thermal resistance, which is defined as follows:

(6) $$R_{g}=\frac{\Delta T}{Q}$$

In particular, such a parameter is composed of the sum of two contributions, which are the thermal contact resistance *R_c_* and the thermal convective resistance *R_h_*:

(7) $$R_{g}=R_{c}+R_{h}$$

**Figure 10:**
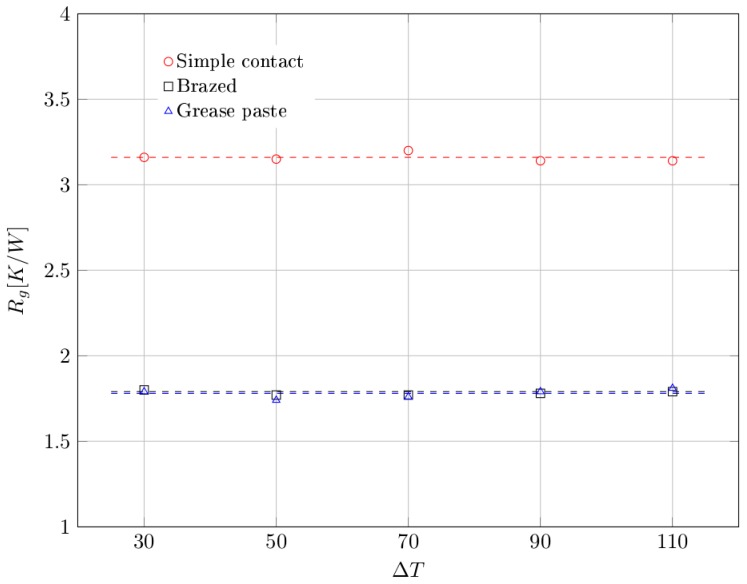
Global thermal resistance as a function of differential temperature, for each type of contact. Dashed lines represent the average values.

Figure 10 shows the global thermal resistance trends obtained through our experimental setup, for the three cases analyzed, as a function of the differential temperature imposed.

Similarly to the global heat transfer coefficient, the global thermal resistance varies depending on the type of contact adopted. In particular, no substantial differences exist between the case of brazed and grease paste contacts, for which an average value of *R_g_* = 1.8 K/W was found. On the contrary, for the simple contact case we report a much higher average value of global thermal resistance (*R_g_* = 3.2 K/W).

### Thermal Contact Resistance Evaluation

In order to understand the impact of each contribution composing the global thermal resistance and to estimate the value of the TCR, the following model is proposed.

Referring to $R_{g1}$, $R_{g2}$ and $R_{g3}$ as the global thermal resistances related to the cases of brazing, grease paste and simple contact, respectively, it is possible to decompose each of such quantities as follows:

(8) $$R_{g1}=R_{brazing}+R_{h1}$$

(9) $$R_{g2}=\frac{R_{paste}\;TCR}{R_{paste}+TCR}+R_{h2}$$

(10) $$R_{g3}=TCR+R_{h3}$$

In the set of Equations (8)–(10) we distinguish the contribution of $R_{c}$ by adopting a different nomenclature depending on the nature of the thermal contact resistance. Therefore, in relations (9) and (10), it is indicated with TCR the thermal contact resistance associated with the pure contact between foam and hot plate. With $R_{brazing}$ and $R_{paste}$, in Equations (8) and (9), it is indicated, instead, the thermal contact resistance contribution due to the presence of a brazing layer and a grease paste layer, between foam and hot plate, respectively. In particular, adopting an electrical analogy, it is possible to assume that the resistance related to grease paste and pure contact are connected in parallel. On the contrary, since the brazing alloy is concentrated in the regions around the metal foam pins, we assume such a resistance to be connected in series with that associated to the pure contact.

Moreover, the resistance $R_{paste}$ can be evaluated as follows:

(11) $$R_{paste}=\frac{t}{k_{paste}A_{b}}$$

where t represents the reference thickness of the grease paste layer. By considering that, as already mentioned in Section 2.1, 3 g of paste were used, the estimated value for the layer thickness is approximately equal to 0.5 mm. Although the grease paste is uniformly spread on the metal foam support, we remark that this value represents only an estimation, since unavoidable non-uniformities occur during the assembly procedure. With the thickness of grease paste layer defined, it is then possible to evaluate the resistance contribution $R_{paste}$ through Equation (11), which results to be equal to 0.04 K/W.

By assuming that the contribution due to convective resistance is the same for all the cases analyzed, namely $R_{h1}=R_{h2}=R_{h3}=R_{h}$ , we can then solve system of Equations (8)–(10), where the three unknown are $R_{brazing}$, $R_{h}$ and TCR. As a result, we obtain:

(12) $$R_{brazing}≅R_{paste}$$

(13) $$R_{h}≅1.76\;K/W$$

(14) TCR≅1.44 K/W

**Figure 11:**
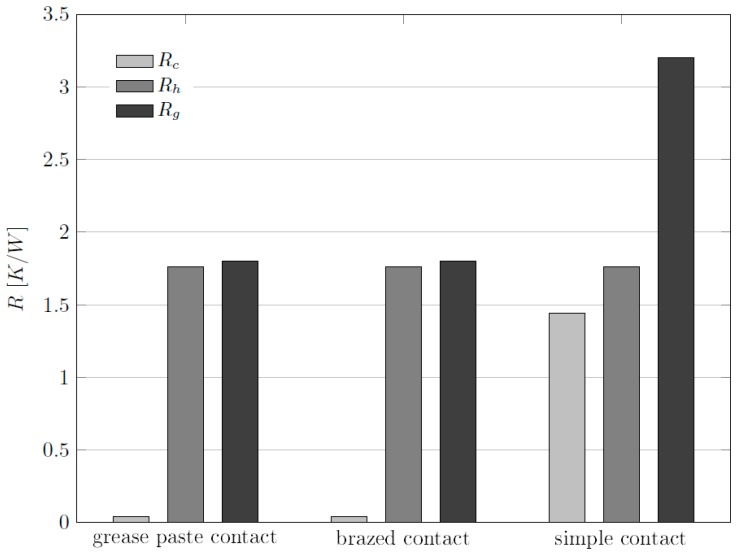
Comparative diagram for the global thermal resistance *R_g_* related to different types of contact. The global thermal resistance is expressed as the sum of two contributions: thermal contact resistance *R_c_* and thermal convective resistance *R_h_*.

The obtained results are reported together with the values of the global thermal resistance in the diagram representation of Figure 11.

The thermal contact resistance associated to the simple contact solution is significantly higher than those related to brazed and grease paste contact. Its value is indeed comparable to that related to the convective resistance, for the same type of contact. On the contrary, the thermal contact resistance for the cases of brazing and grease paste is negligible with respect to the global thermal resistance. On the light of these considerations, it is possible to conclude that the dependence of the global thermal resistance from the type of contact is leaded mainly by the effect of the contact resistance *R_c_*. In particular, the results show that the simple contact is less efficient, in terms of heat transfer performance, than the other two types of contact considered.

## 5. Conclusions

In this study, an experimental analysis on high porosity aluminum open-cell foams in forced convection was carried out. In particular, the effects, in terms of heat transfer performances, due to the thermal contact resistance, for a wide range of differential temperature between sample base and external ambient were investigated. Three different types of contacts between the sample and the heated plate were analyzed: simple contact, brazed contact and grease paste contact.

As expected, the measured global heat transfer coefficient was found to be not dependent from the differential temperature imposed between the metal foam and the ambient, for each case of contact considered. Results show that the simple contact solution is the less efficient, since the average global heat transfer coefficient evaluated for this case is the lowest. On the contrary, higher thermal performances can be achieved for a metal foam by ensuring a brazed or a grease paste contact. In particular, our analysis shows that no substantial differences exist between the case of brazed contact and grease paste contact, in terms of heat transfer performances. The global heat transfer coefficient for these two cases was found to be almost the double than the one obtained for the simple contact case.

## Figures and Tables

**Table 1:** Geometrical and physical parameters for the employed aluminum foam samples.

Parameter	Symbol	Value
Fibre diameter	*d_f_*	5 × 10^−4^ [m]
Pore diameter	*d_p_*	4 × 10^−3^ [m]
Porosity	*ε*	0.965
Permeability	*K*	2.7 × 10^−7^ [m^2^]
Pores per inch	*PPI*	5

**Table 2:** Experimental values of dissipated heat power as a function of differential temperature.

ΔT	Dissipated Heat Q [W]		
	Simple Contact	Brazed	Grease Paste
30	9.49	16.64	16.80
50	15.89	28.17	28.71
70	21.85	39.45	39.85
90	28.67	50.45	50.40
110	35.04	61.38	60.66

**Table 3:** Experimental values of global heat transfer coefficient as a function of differential temperature.

ΔT	Global Heat Transfer Coefficient U [W/m^2^K]		
	Simple Contact	Brazed	Grease Paste
30	126.50	221.81	223.97
50	127.13	225.83	229.65
70	125.83	225.45	227.72
90	127.40	224.22	224.02
110	127.21	223.18	220.58
Mean	127.81	224.10	225.19

## Nomenclature

$A_{b}$	base area	m^2^
$d_{f}$	fibre diameter	m
$d_{p}$	pore diameter	m
ΔT	$\left(T_{b}-T_{amb}\right)$	K
e	error function	K
K	permeability	m^2^
$k_{f}$	air thermal conductivity	Wm^−1^K^−1^
Nu	Nusselt number	
PPI	pores per inch
Q	heat	W
$R_{c}$	thermal contact resistance	KW^−1^
$R_{g}$	global thermal resistance	KW^−1^
$R_{h}$	thermal convective resistance	KW^−1^
$T_{amb}$	ambient temperature	K
$T_{b}$	base temperature	K
$T_{p}$	heated plate temperature	K
U	global heat transfer coefficient	Wm^−2^K^−1^
$u_{i}$	measurement uncertainty of the quantity *i*	[i]
$u_{i\%}$	relative measurement uncertainty of the quantity *i*	
Greek symbols		
ε	porosity	

## References

[1] Kim S., Lee C.W. (2014). A Review on Manufacturing and Application of Open-cell Metal Foam. Procedia Mater. Sci..

[2] Banhart J. (2001). Manufacture, characterisation and application of cellular metals and metal foams. Prog. Mater. Sci..

[3] Zhao C.Y. (2012). Review on thermal transport in high porosity cellular metal foams with open cells. Int. J. Heat Mass Transf..

[4] Mahjoob S., Vafai K. (2008). A synthesis of fluid and thermal transport models for metal foam heat exchangers. Int. J. Heat Mass Transf..

[5] Barletta M., Guarino S., Montanari R., Tagliaferri V. (2007). Metal foams for structural applications: Design and manufacturing. Int. J. Comput. Integr. Manuf..

[6] Barletta M., Gisario A., Guarino S., Rubino G. (2009). Production of open cell aluminum foams by using the dissolution and sintering process (DSP). J. Manuf. Sci. Eng. Trans. ASME.

[7] Guarino S., Barletta M., Pezzola S., Vesco S. (2012). Manufacturing of steel foams by Slip Reaction Foam Sintering (SRFS). Mater. Des..

[8] Antenucci A., Guarino S., Tagliaferri V., Ucciardello N. (2015). Electro-deposition of graphene on aluminium open cell metal foams. Mater. Des..

[9] Bhattacharya A., Calmidi V.V., Mahajan R.L. (2002). Thermophysical properties of high porosity metal foams. Int. J. Heat Mass Transf..

[10] Calmidi V.V., Mahajan R.L. (2000). Forced Convection in High Porosity Metal Foams. J. Heat Transf..

[11] Bai M., Chung J.N. (2011). Analytical and numerical prediction of heat transfer and pressure drop in open-cell metal foams. Int. J. Therm. Sci..

[12] Amiri A., Vafai K., Kuzay T.M. (1995). Effects of Boundary Conditions on Non-Darcian Heat Transfer Through Porous Media and Experimental Comparisons. Numer. Heat Transf. Part A Appl..

[13] Vafai K., Sozen M. (1990). Analysis of Energy and Momentum Transport for Fluid Flow through a Porous Bed. J. Heat Transf..

[14] Amiri A., Vafai K. (1994). Analysis of dispersion effects and non-thermal equilibrium, non-Darcian, variable porosity incompressible flow through porous media. Int. J. Heat Mass Transf..

[15] Zhao C.Y., Lu T.J., Hodson H.P. (2005). Natural convection in metal foams with open cells. Int. J. Heat Mass Transf..

[16] Guarino S., Rubino G., Tagliaferri V., Ucciardello N. (2015). Thermal behavior of open cell aluminum foams in forced air: Experimental analysis. Meas. J. Int. Meas. Confed..

[17] Bhattacharya A., Mahajan R.L. (2006). Metal Foam and Finned Metal Foam Heat Sinks for Electronics Cooling in Buoyancy-Induced Convection. J. Electron. Packag..

[18] Barbieri M., Di Ilio G., Patanè F., Bella G. (2017). Experimental investigation on buoyancy-induced convection in aluminum metal foams. Int. J. Refrig..

[19] Kim S.Y., Kang B.H., Kim J.H. (2001). Forced convection from aluminum foam materials in an asymmetrically heated channel. Int. J. Heat Mass Transf..

[20] Mancin S., Zilio C., Diani A., Rossetto L. (2013). Air forced convection through metal foams: Experimental results and modeling. Int. J. Heat Mass Transf..

[21] Boomsma K., Poulikakos D., Zwick F. (2003). Metal foams as compact high performance heat exchangers. Mech. Mater..

[22] Zhao C.Y., Lu W., Tassou S.A. (2006). Thermal analysis on metal-foam filled heat exchangers. Part II: Tube heat exchangers. Int. J. Heat Mass Transf..

[23] Kim S.Y., Paek J.W., Kang B.H. (2000). Flow and heat transfer correlations for porous fin in a plate-fin heat exchanger. J. Heat Transf..

[24] Huisseune H., De Schampheleire S., Ameel B., De Paepe M. (2015). Comparison of metal foam heat exchangers to a finned heat exchanger for low Reynolds number applications. Int. J. Heat Mass Transf..

[25] Hsieh W.H., Wu J.Y., Shih W.H., Chiu W.C. (2004). Experimental investigation of heat-transfer characteristics of aluminum-foam heat sinks. Int. J. Heat Mass Transf..

[26] Lu W., Zhao C.Y., Tassou S.A. (2006). Thermal analysis on metal-foam filled heat exchangers. Part I: Metal-foam filled pipes. Int. J. Heat Mass Transf..

[27] Ribeiro G.B., Barbosa J.R., Prata A.T. (2012). Performance of microchannel condensers with metal foams on the air-side: Application in small-scale refrigeration systems. Appl. Therm. Eng..

[28] Nield D.A., Bejan A. (2013). Convection in Porous Media.

[29] Bahrami M., Yovanovich M.M., Culham J.R. (2006). Effective thermal conductivity of rough spherical packed beds. Int. J. Heat Mass Transf..

[30] Sadeghi E., Hsieh S., Bahrami M. (2011). Thermal conductivity and contact resistance of metal foams. J. Phys. D Appl. Phys..

[31] Antenucci A., Guarino S., Tagliaferri V., Ucciardello N. (2014). Improvement of the mechanical and thermal characteristics of open cell aluminum foams by the electrodeposition of Cu. Mater. Des..

[32] Fiedler T., White N., Dahari M., Hooman K. (2014). On the electrical and thermal contact resistance of metal foam. Int. J. Heat Mass Transf..

[33] Mancin S., Zilio C., Cavallini A., Rossetto L. (2010). Heat transfer during air flow in aluminum foams. Int. J. Heat Mass Transf..

[34] Ghosh I. (2009). Heat transfer correlation for high-porosity open-cell foam. Int. J. Heat Mass Transf..

